# Chitosan nanocomposite containing rotenoids: an alternative bioinsecticidal approach for the management of *Aedes aegypti*

**DOI:** 10.3762/bjnano.16.88

**Published:** 2025-07-28

**Authors:** Maria A A Bertonceli, Vitor D C Cristo, Ivo J Vieira, Francisco J A Lemos, Arnoldo R Façanha, Raimundo Braz-Filho, Gustavo V T Batista, Luis G M Basso, Sérgio H Seabra, Thalya S R Nogueira, Felipe F Moreira, Arícia L E M Assis, Antônia E A Oliveira, Kátia V S Fernandes

**Affiliations:** 1 Laboratório de Química e Função de Proteinas e Peptídeos, CBB, Universidade Estadual do Norte Fluminense Darcy Ribeiro, Campos dos Goytacazes/RJ, Brazil; 2 Laboratório de Ciências Químicas, CCT, Universidade Estadual do Norte Fluminense Darcy Ribeiro, Campos dos Goytacazes/RJ, Brazil; 3 Laboratório de Biotecnologia, CBB, Universidade Estadual do Norte Fluminense Darcy Ribeiro, Campos dos Goytacazes/RJ, Brazil; 4 Laboratório de Biologia Celular e Tecidual, CBB, Universidade Estadual do Norte Fluminense Darcy Ribeiro, Campos dos Goytacazes/RJ, Brazil; 5 Laboratório de Ciências Físicas, CCT, Universidade Estadual do Norte Fluminense Darcy Ribeiro, Campos dos Goytacazes/RJ, Brazil

**Keywords:** dengue, nanoparticle, pest management, phytochemicals

## Abstract

Climate change has intensified the proliferation of disease vectors, such as *Aedes aegypti*, the primary transmitter of dengue, chikungunya, and zika viruses. Although the two recently licensed dengue vaccines represent a significant advancement, vector management remains the primary strategy for preventing these urban arboviruses. In this context, the development of pesticides that offer safer alternatives for the environment and human health has become urgent. In this study, a chitosan-based nanocomposite was developed as a delivery system for rotenoids isolated from *Clitoria fairchildiana* seeds, leveraging their larvicidal activity against third-instar larvae of *Ae. aegypti*. The nanocomposite was synthesized using a controlled ionic gelation method incorporating the TPP-β-CD inclusion complex, which resulted in nanoparticles with smaller size, improved polydispersity index, and enhanced stability, evidenced by a higher zeta potential. FTIR analysis confirmed rotenoid incorporation into the nanocomposite and suggested hydrogen bonding or potential covalent interaction with chitosan functional groups. Bioassays demonstrated that the nanocomposite achieved an LC_50_ of 91.7 ppm, representing a 23.6% increase in larvicidal efficacy compared to the rotenoids in their natural form. The nanocomposite also induced dose-dependent morphological and physiological alterations in the larvae, including damage to the peritrophic matrix, evidenced by abnormal anal excretion, and tissue melanization and formation of melanotic pseudotumors. These responses may be associated with increased production of reactive oxygen species in the larval midgut, consistent with previous findings for the nonencapsulated rotenoids. Importantly, empty nanoparticles exhibited no adverse effects on larval survival, which is attributed to the biocompatibility and nontoxic nature of chitosan, a biodegradable polysaccharide structurally related to the insect exoskeleton and widely recognized for its environmental safety. Additionally, neither rotenoids nor the CS/TPP–β-CD–rot nanocomposite exerted cytotoxic effects, confirming their favorable safety profile. These findings highlight the potential of nanotechnology to enhance the efficacy of bioactive compounds while minimizing environmental and human health risks, offering a sustainable and innovative strategy for vector control.

## Introduction

Climate change has significantly impacted public health, intensifying the proliferation of disease vectors such as those transmitted by the mosquito *Aedes aegypti*. Environmental conditions exacerbated by global warming, including rising temperatures, heavy rainfalls, and high humidity, have accelerated mosquito reproduction and expanded its geographic spread. In 2023, dengue reached a record 5 million cases in 80 countries, including historically non-endemic regions such as the United States, Italy, France, and Spain. Recent studies indicate that the risk of dengue transmission has increased by 11% in the past decade due to the climate crisis [1]. From even more recent data from Brazil for the year 2024, dengue cases increased by 297%, and chikungunya cases by 65% compared to the same period in 2023. These figures include 5,696 deaths from dengue and 188 deaths from chikungunya. Additionally, while no Zika cases were reported in 2023, 6,348 cases were recorded in 2024 [2]. All these alarming numbers underscore the urgent need for sustainable and innovative strategies to manage *Ae. aegypti* and mitigate the effects of this increasingly global and urgent threat.

According to the World Health Organization (WHO), two dengue vaccines have been licensed: Dengvaxia (CYD-TDV), developed by Sanofi Pasteur, and Qdenga (TAK-003), developed by Takeda. CYD-TDV is a live recombinant tetravalent dengue vaccine approved for use in individuals aged 9–45 years or 9–60 years, depending on country-specific regulatory guidelines. Due to the requirement for pre-vaccination screening, the widespread use of this vaccine has been limited. The use of TAK-003 is recommended for children aged 6 to 16 years in regions with high dengue transmission intensity. However, its use is not currently recommended for children under 6 years of age or adults aged 60 years and older, due to the lower effectiveness of the vaccine in these age groups [3]. While these vaccines represent significant advancements in dengue prevention, *Ae. aegypti* vector management remains the primary strategy for controlling dengue and other urban arboviruses, such as chikungunya and zika.

While most places are increasingly investing in the application of larger quantities of synthetic larvicides, such as the unprecedented investment of 16.9 million Brazilian reais in 2023 [4], the development of resistance by this vector to regularly used larvicides, which impact other organisms beneficial to the environment while presenting risks of poisoning to humans who handle them, is rampant. The adoption of new biotechnological tools becomes crucial to overcome these obstacles in vector control in a smarter way [5–8].

Bioactive compounds from plants have demonstrated great potential as bioinsecticidal agents. Derived from renewable sources, these compounds possess complex chemical compositions, diverse modes of action, and selective toxicity to target organisms, making them a viable alternative for developing new insecticide formulations. Combining these attributes with nanoparticle encapsulation strategies, it is possible to considerably increase the biocidal agent efficacy while reducing the environmental and human health impacts associated with traditional synthetic insecticides [9–10].

Chitosan nanoparticles, derived from a biodegradable and nontoxic polysaccharide, have proven effective in reducing post-harvest deterioration of fruits and vegetables, in addition to exhibiting well-documented antimicrobial properties [11–12]. Furthermore, a chitosan nanocomposite with fungal metabolites has been reported as a promising alternative for managing insect pests of economic importance without affecting non-target organisms [13].

In previous research by our group, two rotenoids were identified and characterized from *Clitoria fairchildiana* seeds with larvicidal activity against third-instar *Ae. aegypti* larvae. These studies revealed morphological and metabolic changes in the larvae, suggesting that V-ATPase inhibition triggers oxidative stress, resulting in high production of reactive oxygen species (ROS) in the midgut. This process causes exoskeletal alterations and ultimately leads to larval death [14].

Building on this work and aiming to develop a biotechnological product that could represent a novel tool for managing *Ae. aegypti*, a chitosan (CS)-based nanocomposite was developed as a delivery system for the isolated rotenoids. The nanocomposite represented a 23.6% increase in efficacy compared to the previously described natural rotenoids. These results highlight the potential of chitosan nanocomposites as an innovative, effective, and environmentally sustainable solution for managing the insect vector. By offering a promising alternative, this technology directly contributes to preventing diseases such as dengue, zika, and chikungunya.

## Results and Discussion

Two rotenoids, identified as 11α-*O*-β-ᴅ-glucopyranosylrotenoid (CFD – RI) and 6-deoxyclitoriacetal *O*-β-ᴅ-glucopyranoside (CFD – RII), were previously isolated and characterized from *C. fairchildiana* seeds [14]. These compounds exhibited larvicidal activity against third-instar *Ae. aegypti* larvae, causing morphological changes and metabolic alterations as a result of increased production of ROS in the insect midgut [14]. It is already well-documented in the literature that larvicidal bioassays are effective tests for identifying new insecticides against this vector mosquito, as the larval stage is the longest and most vulnerable immature phase of the insect. In addition, this species is synanthropic, which means that it is adapted to live close to humans who, in their life routine, create spaces of easy access and conducive conditions for the establishment of the insect. The most frequent breeding sites are containers with accumulated water, such as plant pots, PET bottles, tires, buckets, clogged or uneven gutters, rarely used drains, poorly sealed water tanks, air conditioner trays, and other locations with stagnant and "clean" water containing organic matter [15].

In this work, with the goal of obtaining a biotechnological product that may represent a new tool for controlling *Ae. aegypti* and consequently preventing relevant arboviral diseases, we produced a chitosan nanocomposite with the isolated rotenoids.

Chitosan is an aminated polysaccharide derived from the deacetylation of chitin and is the main structural component of insect and crustacean exoskeletons. It is widely recognized as the second most abundant natural polymer, surpassed only by cellulose. Its relevance as a biopolymer is due to its properties such as biodegradability, biocompatibility, and nontoxicity to humans, characteristics that make it widely used in various scientific and technological fields [16–17]. Therefore, we used chitosan as the coating material for the nanocomposite to reduce potential environmental damage, such as those reported in the literature for some metal-decorated nanomaterials and their derivatives (silver, gold, copper, zinc, titanium, and silicon) that can be highly toxic to non-target organisms in the environment [18].

**Figure 1:**
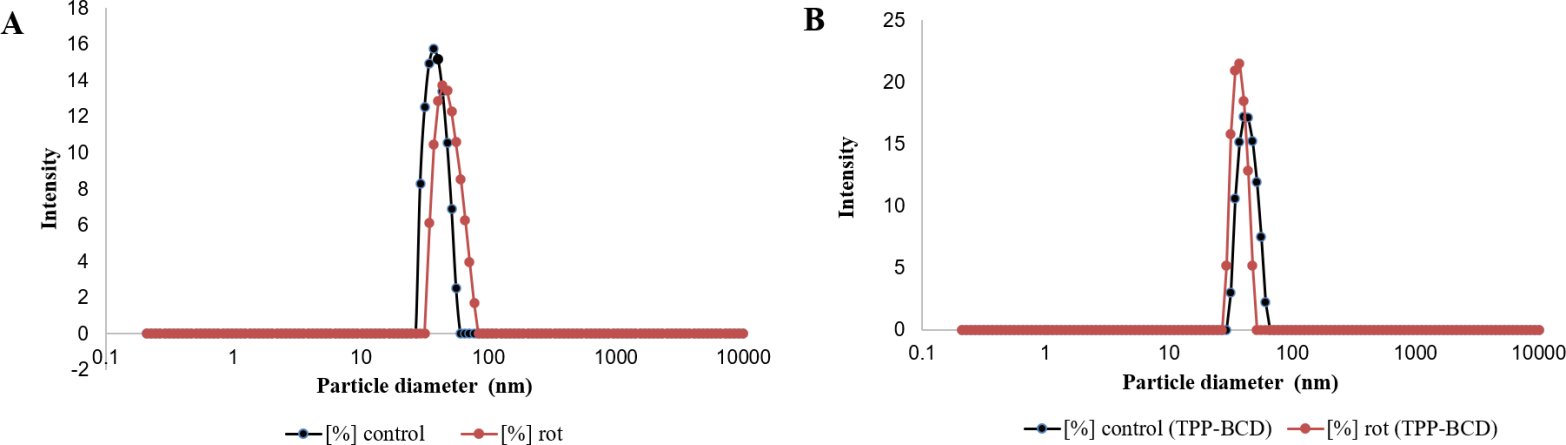
Size distribution and zeta potential of nanoparticles. A) Conventional ionic gelation of CS/TPP (control = empty particles; rot = rotenoid-loaded particles). B) Controlled ionic gelation of CS with TPP-β-CD inclusion complex (control and rot).

**Figure 2:**
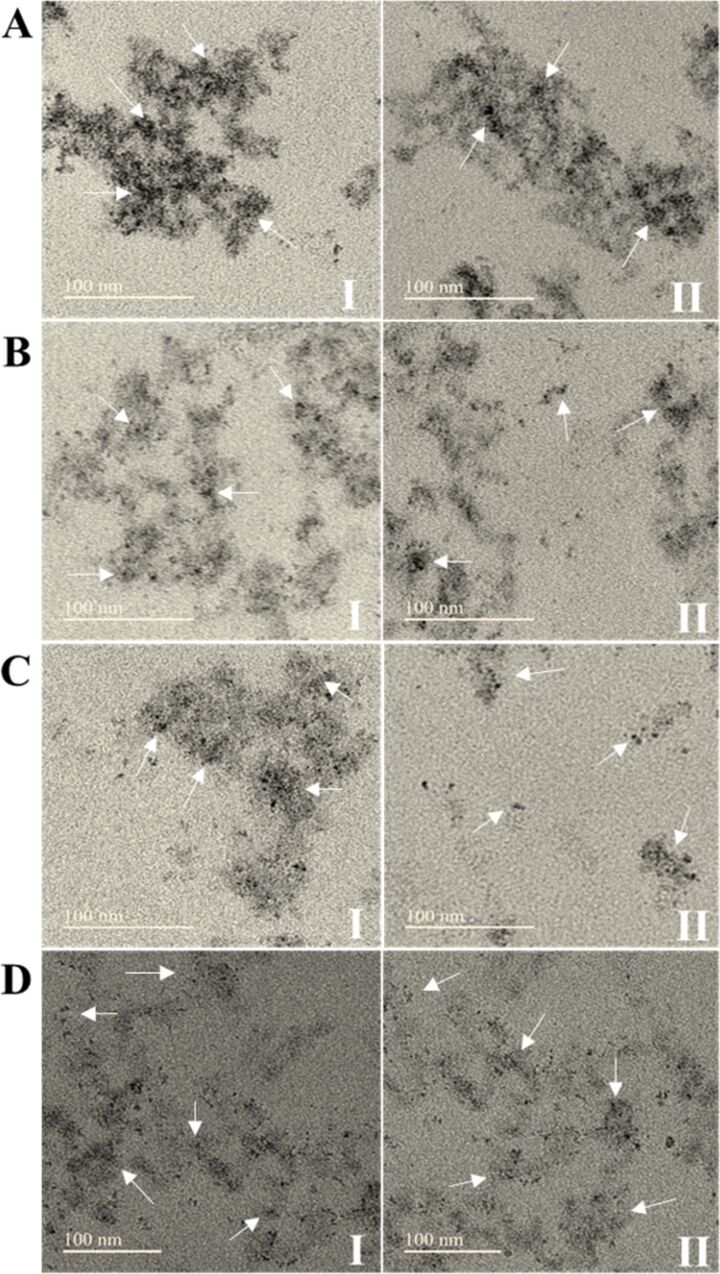
Transmission electron microscopy (TEM) of chitosan nanocomposites. A) Conventional ionic gelation of CS/TPP. B) Conventional ionic gelation of CS/TPP containing rotenoids (CFD – RI and RII). C) Controlled ionic gelation of CS with TPP-β-CD inclusion complex. D) Controlled ionic gelation of CS with TPP-β-CD inclusion complex containing rotenoids (CFD – RI and RII).

The encapsulation efficiency is estimated to be ≥99% within the detection limits of high-performance liquid chromatography (HPLC) and thin-layer chromatography (TLC) methods applied, indicating a highly effective incorporation of the rotenoids into the nanochitosan matrix. In addition to the high encapsulation efficiency, the size analysis of the produced nanochitosan particles confirmed that both ion gelation methods produced nanostructures smaller than 100 nm (Figure 1). However, in Figure 1B, the peak area is narrower, indicating a higher uniformity in particle size. Figure 2C and 2D also show that the controlled ionic gelation method using the TPP-β-CD inclusion complex resulted in smaller and more uniform nanoparticles compared to the conventional ionic gelation method (CS/TPP), illustrated in Figure 2A. This improvement can be attributed to the ability of the TPP-β-CD inclusion complex to mitigate excessive cross-linking by TPP, thereby reducing both nanoparticle size and polydispersity [19]. Furthermore, the inclusion of rotenoids in the nanocomposite seemingly improved its dispersibility (Figure 2B and 2D) compared to respective controls of empty nanoparticles (Figure 2A and 2C). The presence of rotenoids in the nanostructures also increased the zeta potential (ζ) in both ion gelation methods, which appears to influence the polydispersity index (PDI) of these nanocomposites, as the PDI values are significantly higher than those of their respective controls (Table 1). The zeta potential is an important parameter for assessing nanoparticle stability and biodistribution. Typically, particles acquire an electric charge at the shear plane when dispersed in a liquid, which is reflected by their zeta potential, that is key to understanding dispersion and aggregation processes in nanoformulations.

**Table 1:** DLS analysis of chitosan nanocomposites. Average particle diameter (*d*), polydispersity index, and average zeta potential (mV) obtained by DLS.

	Samples	*d* (nm)	PDI (%)	ζ (mV)

A -	CS/TPP control	32.9	10.4	36.3
B -	CS/TPP rot	41.5	21.2	57.1
C -	CS/TPP - βCD control	35.1	21.1	44.8
D -	CS/TPP - βCD rot	29.5	27.0	51.7

Zeta potential values > ±30 mV are indicative of nanoparticle stability, as strong electrostatic repulsion prevents aggregation and ensures colloidal stability [20–21]. While these measurements provide important physicochemical insights, further evidence of the chemical incorporation and potential interactions between the rotenoids and the nanocomposite matrix was obtained through Fourier-transform infrared (FTIR) spectroscopy.

**Figure 3:**
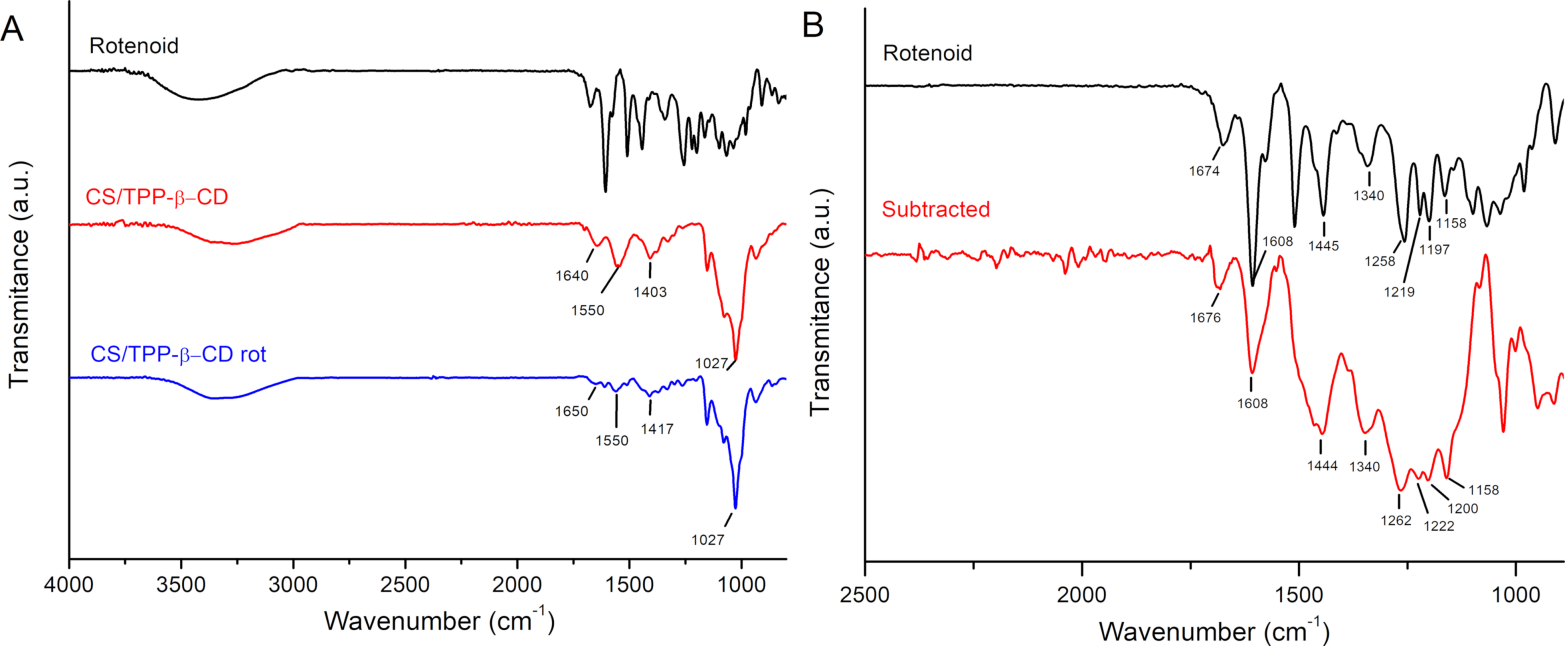
FTIR analysis of pure rotenoids and chitosan-based nanoparticles. A) FTIR spectra of pure rotenoids, empty nanoparticles (CS/TPP-β-CD), and rotenoid-loaded nanoparticles (CS/TPP-β-CD-rot). B) Comparison between the spectrum of in natura rotenoids and the differential spectrum obtained by subtracting the CS/TPP-β-CD spectrum from that of CS/TPP-β-CD-rot, highlighting the characteristic absorption bands of the incorporated rotenoids.

Figure 3A shows the FTIR spectra of CS/TPP-β-CD, CS/TPP-β-CD loaded with rotenoids (CS/TPP-β-CD-rot), and pure rotenoids. All three spectra display a broad absorption band in the 3600–3000 cm^−1^ region, corresponding to the stretching vibrations of hydroxyl and amide groups. In the nanoparticle spectra, characteristic bands are observed at 1640–1650 cm^−1^ and around 1550 cm^−1^, associated with amide I (C=O stretching) and amide II (N–H bending), respectively. Peaks in the 1417–1403 cm^−1^ range are attributed to the bending vibrations of C–H in methyl and methylene groups [22–24]. The spectrum of pure rotenoids displays a complex profile with multiple peaks due to the presence of diverse functional groups [14]. Notably, 6-aromatic C=C stretching appears in the 1600–1300 cm^−1^ region, while glycosidic linkages and methoxyl groups contribute bands in the 1300–1000 cm^−1^ range [25–26]. Figure 3B presents a spectral subtraction analysis between CS/TPP-β-CD and CS/TPP-β-CD-rot, clearly revealing absorption features from the encapsulated rotenoids in the functionalized nanocomposite. Additionally, a noticeable reduction in the intensity of the amide-related bands in CS/TPP-β-CD-rot suggests possible interactions – such as hydrogen bonding or covalent bonding – between rotenoids and the chitosan matrix. These spectral features confirm the successful incorporation of rotenoids into the chitosan-based nanocomposite and suggest molecular interactions that may contribute to the enhanced stability and bioactivity of the formulation.

**Figure 4:**
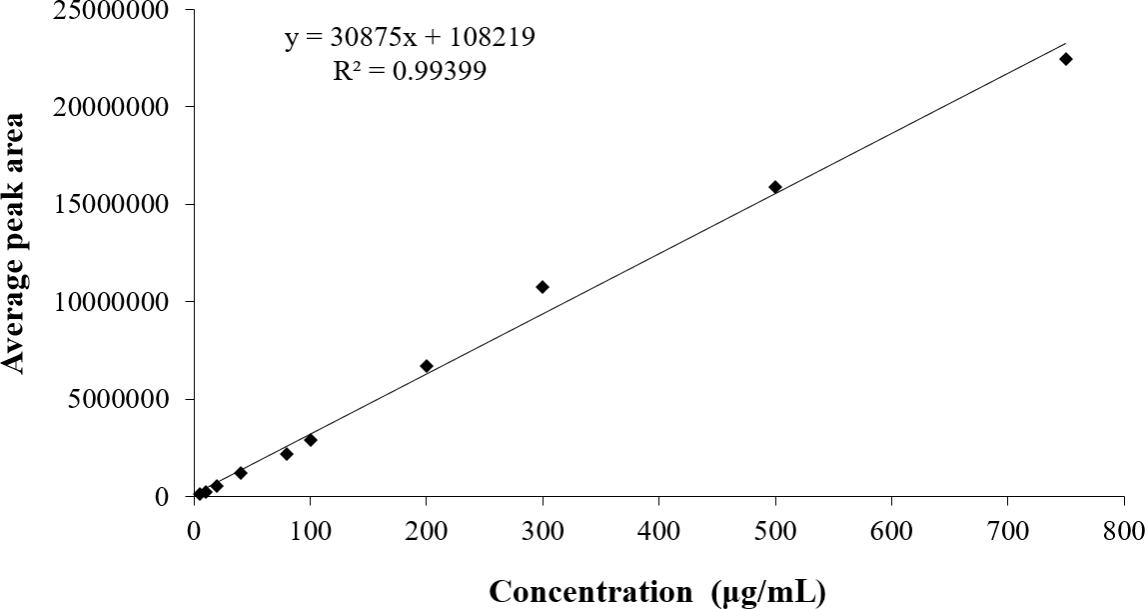
Calibration curve of rotenoids (CFD-RI and RII) by HPLC.

To complement the physicochemical characterization and confirm the efficiency of rotenoid encapsulation within the nanoparticles, a calibration curve was constructed for the raw rotenoids by HPLC using linear regression (Figure 4), enabling the quantification of rotenoids incorporated into the nanostructures. Using this approach, the rotenoid concentration was determined to be 0.469 mg/mL for the conventional ionic gelation method and 0.531 mg/mL for the controlled ionic gelation method.

**Figure 5:**
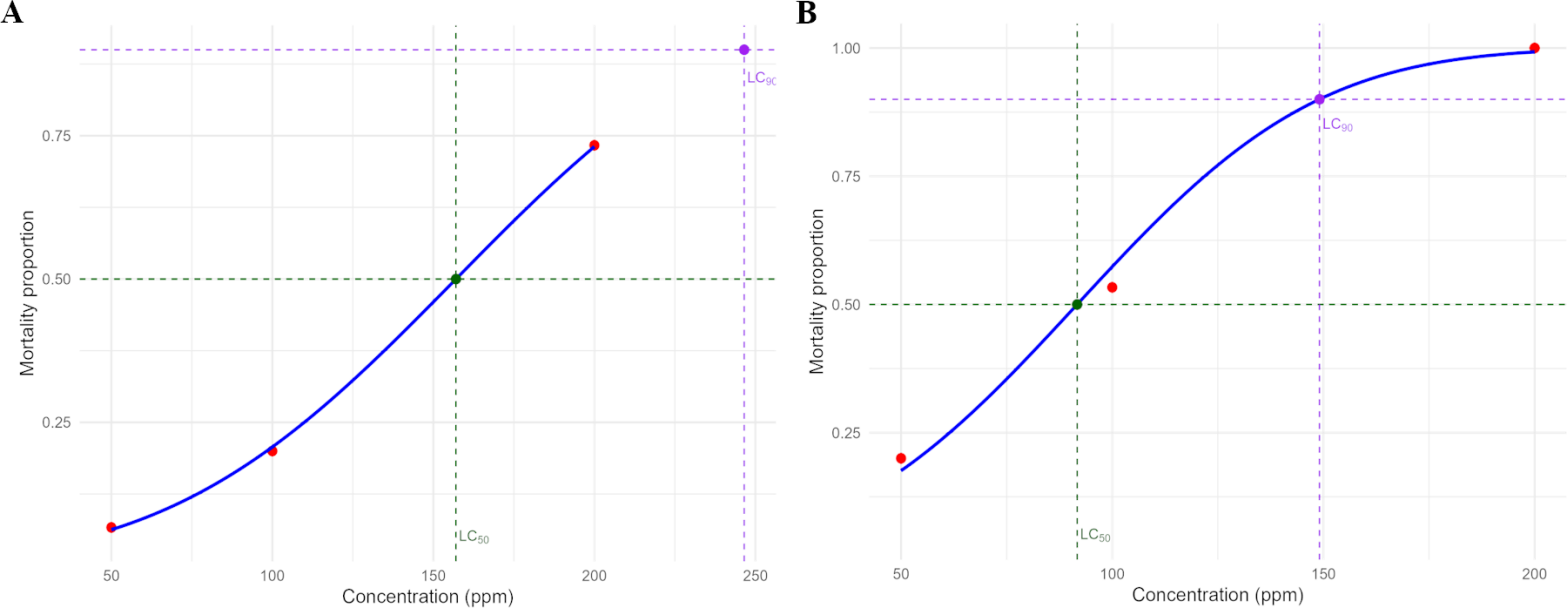
Mortality proportion of third-instar *Aedes aegypti* larvae as a function of concentration (ppm) of nanocomposites containing rotenoids. Negative control – vinegar (0.04% acetic acid) resulted in 0% mortality. A) Conventional ionic gelation (CS/TPP–rot) with LC_50_ = 157 ppm and LC₉₀ = 247 ppm, calculated using Probit analysis performed in RStudio. B) Controlled ionic gelation (CS/TPP–β-CD–rot) with LC_50_ = 91.7 ppm and LC₉₀ = 149 ppm, calculated using Probit analysis performed in RStudio. The blue line represents the fitted Probit model. Red circles represent the observed mortality proportion. The green dashed lines and green marker indicate LC_50_, while the purple dashed lines and purple marker indicate LC_90_. Each treatment was performed with five larvae per concentration in triplicate (*n* = 15 per treatment).

The larvicidal bioassay of the nanocomposite produced by the controlled ionic gelation method (CS/TPP–β-CD–rot, Figure 5B) revealed a significant increase in larvicidal activity, with an LC_50_ of 91.69 ppm and an LC_90_ of 149.06 ppm, indicating higher larvicidal efficacy. In comparison, the in natura rotenoids described earlier exhibited an LC_50_ of 120 ppm [14], demonstrating that the nanocomposite improved the efficacy of the rotenoids by 23.6%. These results were obtained using the classical Probit method recommended by the World Health Organization (WHO) for larvicidal bioassays, which applies the cumulative normal distribution model.

In contrast, the bioassay conducted with the nanocomposite produced by the conventional ionic gelation method (CS/TPP–rot, Figure 5A) showed an LC_50_ of 157.02 ppm and an LC_90_ of 246.52 ppm, a value notably higher than that observed for the in natura rotenoids, indicating a lower potency compared to the CS/TPP–β-CD–rot formulation. Control nanoparticles (CS/TPP and CS/TPP–β-CD) exhibited no larval mortality, confirming that the observed larvicidal activity is directly associated with the rotenoids.

Previous studies have reported similar improvements in larvicidal activity through nanocarrier systems based on botanical insecticides. For instance, silver nanoparticles (AgNPs) synthesized using aqueous leaf extract of *Ambrasia arborescens* demonstrated markedly higher toxicity against *Aedes aegypti* larvae (LC_50_ = 0.28 ppm) compared to the crude aqueous extract (LC_50_ = 1844.61 ppm) [27]. Similarly, silver nanoparticles synthesized with aqueous extracts of *Solanum mammosum* (SmAgNPs) exhibited significantly greater toxicity (LC_50_ = 0.06 ppm) than the crude aqueous extract (LC_50_ = 1631.27 ppm) [28].

According to an established classification criterion, botanical insecticides with an LC_50_ below 50 µg/mL (50 ppm) are considered highly active; those with an LC_50_ between 50 and 100 µg/mL (50–100 ppm) are classified as active; and those with an LC_50_ above 100 µg/mL (100 ppm) are regarded as weak or inactive [9]. Therefore, technically speaking, both *Ambrasia arborescens* and *Solanum mammosum* crude extracts are classified as weak bioactive agents. However, when incorporated into nanostructures, they acquire substantially enhanced insecticidal potential. A similar pattern is observed in this study, where the incorporation of rotenoids into the CS/TPP–β-CD nanocomposite effectively improves their larvicidal activity. Furthermore, nanostructured delivery systems not only enhance the biological efficacy of botanical compounds but also improve their stability under adverse environmental conditions, such as temperature fluctuations and UV light exposure, as widely documented in the literature [29].

**Figure 6:**
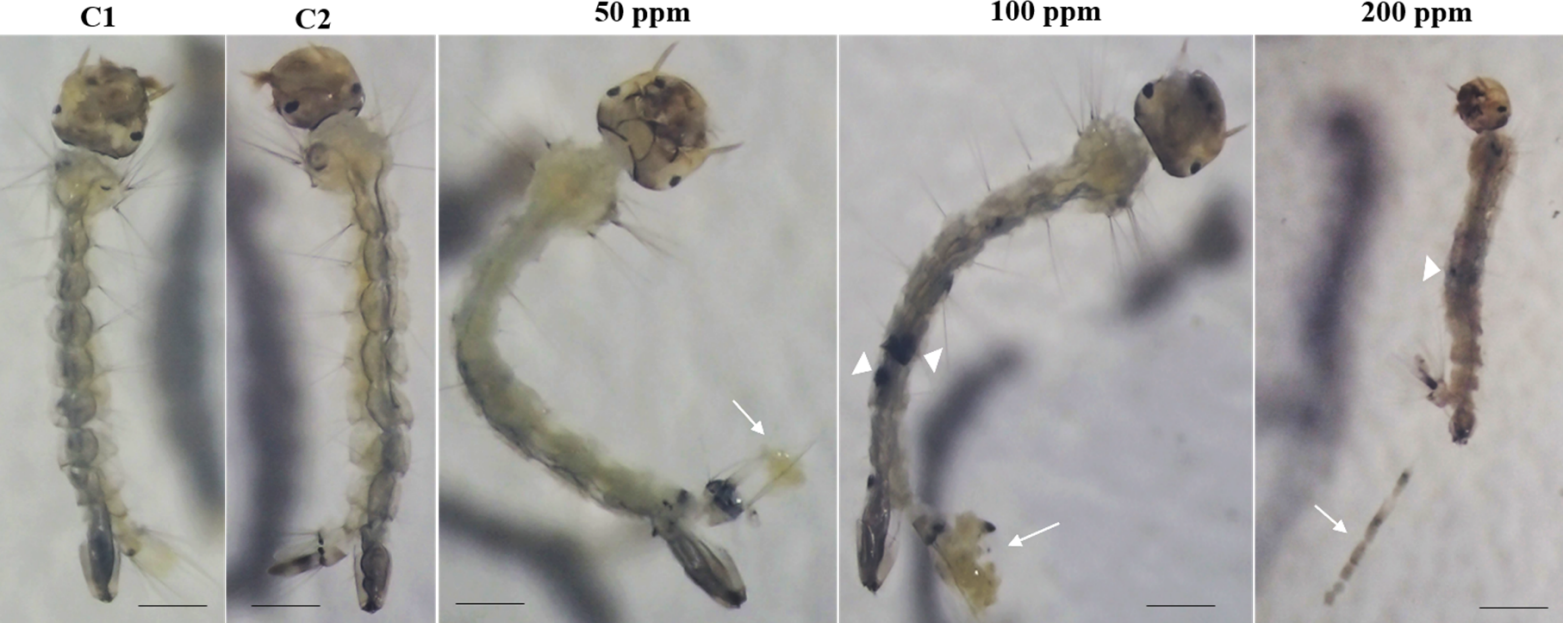
General morphological characteristics of 3rd instar *Aedes aegypti* larvae exposed to different concentrations (ppm) of nanocomposites (CS/TPP-β-CD–rot) observed under a stereomicroscope. C1 – negative control with ultrapure water. C2 – negative control with vinegar (acetic acid 0.04%). White arrows indicate the presence of abnormal excretion in the anal region, suggesting possible damage to the peritrophic matrix. White arrowheads point to melanized nodular structures (pseudotumors), indicative of immune activation through the phenoloxidase cascade. Scale bar = 1 mm.

The analysis of third-instar *Ae. aegypti* larvae exposed to the nanocomposite CS/TPP–β-CD–rot (Figure 6) revealed notable dose-dependent morphological alterations. From 50 ppm onwards, the appearance of abnormal excretion in the anal region was observed (Figure 6, white arrows), which progressively intensified at higher concentrations (100 and 200 ppm). This phenomenon may be indicative of physiological stress related to the integrity of the peritrophic matrix (PM), a semi-permeable structure that lines the midgut, playing essential roles in protecting the epithelium, facilitating digestion, and acting as a barrier against pathogens and toxic compounds [30–31].

Similar responses have been documented in previous studies, where resistant *Ae. aegypti* strains exposed to dichlorodiphenyltrichloroethane (DDT) produced abundant PM filaments to aid in the excretion of unabsorbed insecticides, unlike susceptible strains that released little or no PM [32]. Additionally, amorphous feces were reported in larvae fed with *Derris urucu* extracts (rich in rotenoids), suggesting a potential link between exposure to rotenoid-type phytochemicals and PM disruption [33].

Notably, from 100 ppm onwards, larvae exhibited melanized nodular structures interpreted as pseudotumors (Figure 6, white arrow head), a typical outcome of the activation of the phenoloxidase cascade. This immune response is frequently triggered by gut damage, epithelial barrier disruption, or the presence of damage-associated molecular patterns (DAMPs) [34–35]. Melanization serves as a fundamental defense mechanism in arthropods, contributing to the encapsulation of damaged tissues and neutralization of harmful agents, including ROS and foreign particles [36].

It is important to highlight that this tissue melanization observed in the larval midgut aligns with our previous findings, which reported the appearance of dark spots along the larval body associated with oxidative stress induced by in natura rotenoids [14]. In that study, the purified rotenoids triggered significant ROS production, causing metabolic and morphological damage particularly in the midgut, activating immune responses such as melanization [37].

The morphological alterations observed here, including abnormal excretion and tissue melanization, suggest that the nanoencapsulated rotenoids maintain, and even amplify, their ability to damage gut structures such as the PM. This damage likely contributes to the triggering of immune responses, including melanization and pseudotumor formation. These findings are consistent with the hypothesis that the gut remains the primary target of rotenoid toxicity, even when delivered via nanocomposites.

**Figure 7:**
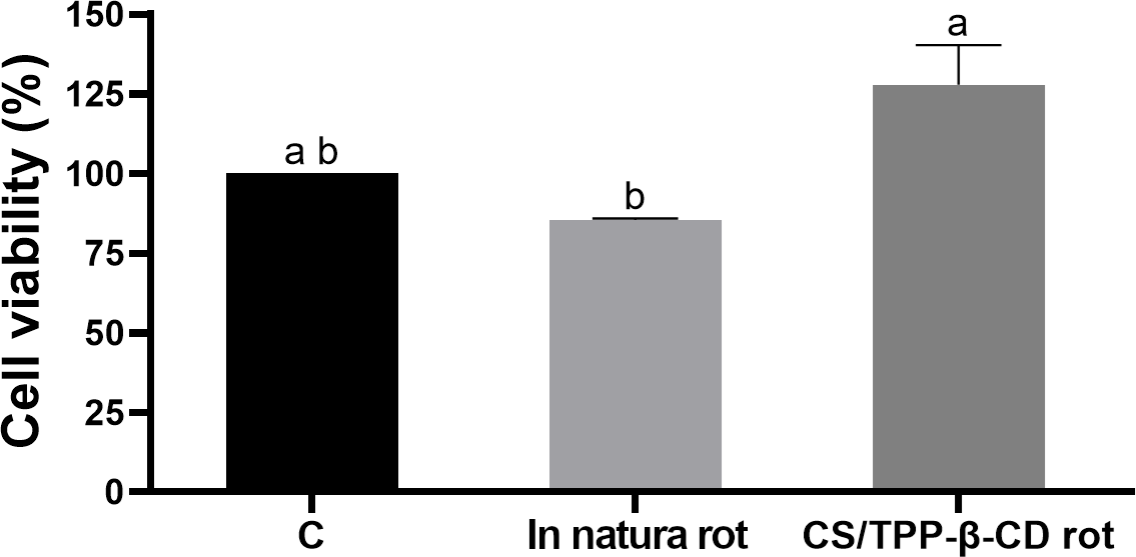
Cytotoxicity of in natura and nanoencapsulated rotenoids (250 ppm) to HSF cells through the MTT assay (3-(4,5-dimethylthiazol-2-yl)-2,5-diphenyltetrazolium bromide) for 24 hours at 37 °C with 5% CO_2_. Statistical analysis was calculated according to ANOVA (Tukey's multiple comparison) (*P* < 0.05).

The cytotoxicity analysis of natural rotenoids to HSF cells revealed no statistically significant differences in cell viability between the control group and the treatments with either in natura rotenoids or the CS/TPP-β-CD–rot nanocomposite (Figure 7; *p* > 0.05). Both treatments maintained cell viability at levels comparable to the control, indicating the absence of cytotoxic effects. These findings demonstrate that the encapsulation of rotenoids into the CS/TPP-β-CD system does not induce cytotoxicity, similarly to the free compound, suggesting that the delivery system is biocompatible with HSF cells. Therefore, both free rotenoids and the CS/TPP-BCD rot formulation are safe for use at the tested concentration. Previous studies reported that synthetic insecticides, such as type II pyrethroids (deltamethrin, cyphenothrin, λ-cyhalothrin, cyfluthrin, esfenvalerate, and α-cypermethrin) can affect cell viability by inducing the production of nitric oxide and lipid peroxides in three human cell models (SH-SY5Y, HepG2, and Caco-2), even at concentrations considered safe [38]. Nevertheless, human exposure to multiple pyrethroids routinely occurs due to the limited regulatory restrictions imposed on the use of these insecticides. The development of pesticides that provide safer alternatives for both the environment and human health is an urgent need [8]. Thus, the nanocomposite developed in this study holds promising potential for the formulation of new pesticides that could serve as an alternative tool for controlling this vector insect.

## Conclusion

In this study, a chitosan-based nanocomposite was developed as a release system for isolated rotenoids, using the controlled ionic gelation method with the TPP-β-CD inclusion complex. This strategy resulted in the production of smaller, more uniform nanoparticles, with better polydispersity indices and increased stability, in comparison to conventional ionic gelation method. FTIR analysis confirmed the incorporation of rotenoids into the nanocomposite and revealed characteristic spectral features that suggest the occurrence of hydrogen bonding or potential covalent interaction with functional groups of chitosan, which may help explain the improved stability of the formulation. The high efficacy of the nanocomposite was demonstrated by bioassays which revealed an LC_50_ of 91.7 ppm, equivalent to a 23.6% improvement in the larvicidal activity rate of the rotenoids in their original forms. The nanocomposites caused morphological and biochemical alterations to the larvae similarly to those caused by in natura rotenoids, but with greater intensity. Cytotoxicity MTT assays using human skin fibroblasts demonstrated that neither rotenoids nor the CS/TPP–β-CD–rot nanocomposite exerted cytotoxic effects, confirming their favorable safety profile. The results obtained confirm that the application of nanotechnology for encapsulating bioactive compounds not only enhances larvicidal efficacy but also contributes to environmentally sustainable solutions by reducing the reliance on synthetic chemical pesticides. By integrating renewable resources such as phytochemicals and chitosan, this work reinforces the potential for the development of green technologies, offering an innovative and promising approach to the management of disease vectors and the prevention of urban arboviral diseases.

## Materials and Methods

### Biological material

#### Aedes aegypti

The larvae of *Ae. aegypti* from the Rockefeller strain were obtained from the insectary maintained at the Biotechnology Laboratory (LBT), at the Center for Biosciences and Biotechnology (CBB) of the State University of North Fluminense Darcy Ribeiro (UENF), where they were raised at room temperature between 20 and 30 °C and fed with fish food.

### Production and characterization of nanocomposites

#### Method of production of chitosan nanocomposites

The nanocomposites for use as a vehicle of the previously isolated and characterized rotenoids [14] were prepared using chitosan (CS Sigma-Aldrich 419419) as the coating material. For comparison purposes and identification of the best method, both common ionic gelation (CS/TPP) and controlled ionic gelation (CS/TPP–β-CD) methods were employed [19], with adaptations in order to include the rotenoid.

In the common ionic gelation method, a 0.175% (w/v) chitosan solution was prepared in 20 mL of 1% (v/v) acetic acid, containing 1 mg/mL of rotenoids. The mixture was maintained under constant agitation at 300 rpm, at room temperature, overnight. Subsequently, 15 mL of a 5 mM sodium tripolyphosphate (TPP) solution was prepared. The chitosan solution was then added dropwise to the TPP solution, while the mixture was continuously agitated on a magnetic stirrer at 1000 rpm, overnight, at room temperature. The resulting solution was centrifuged at 10,000*g*; the pellet was separated, lyophilized, and the obtained powder was stored at −20 °C. To evaluate whether any rotenoids remained unincorporated, the supernatant was also lyophilized and subsequently analyzed by both TLC and HPLC. No rotenoid signals were detected in the supernatant by either method, suggesting that the rotenoids were fully incorporated into the nanocomposites under the conditions employed. The same procedure was applied for the preparation of empty nanoparticles, without the addition of rotenoids. Nanoparticles containing rotenoids were then called rot.

The chitosan nanocomposites were also prepared using the TPP–β-cyclodextrin (TPP–β-CD) inclusion complex. For this, equimolar amounts of TPP and β-CD (β-cyclodextrin) were completely dissolved in ultrapure water at a molar ratio of 1:1. The obtained solution was subjected to ultrasonic bath for 1 h at room temperature. This TPP–β-CD inclusion complex replaced the pure TPP in the controlled ionic gelation process of the chitosan solution, following the same protocol previously described.

In total, four samples were obtained, two for each method. One consisted of empty nanoparticles containing only chitosan (CS/TPP and CS/TPP–β-CD, controls), while the other corresponded to nanocomposites containing rotenoids (CS/TPP rot and CS/TPP–β-CD rot).

#### Size, polydispersity, and zeta potential

The average size (in nm) and PDI of the nanoparticles were measured using dynamic light scattering (DLS) on the Litesizer DLS 500 instrument (Anton Paar). The same equipment was used to determine the zeta potential (ζ, in mV) of the nanoparticles under the following conditions: 25 °C operating temperature, an applied voltage of 200 V, and a total of 1,000 processed runs.

#### Fourier-transform infrared spectroscopy

The chemical structure of empty nanoparticles (CS/TPP-β-CD), pure rotenoids, and rotenoid-loaded nanoparticles (CS/TPP-β-CD-rot) was analyzed using a PerkinElmer Spectrum Two FTIR spectrometer equipped with an Attenuated Total Reflectance (ATR) accessory. Dried samples were scanned over a wavenumber range of 800–4000 cm^−1^, with a resolution of 1 cm^−1^ and an accumulation of 35 scans per spectrum. A compressive force of 15 N was applied to the samples during measurement to ensure proper contact with the ATR crystal surface.

#### Quantification of rotenoids by high-performance liquid chromatography

The quantification of rotenoids was performed by HPLC using a SHIMADZU CBM-20A system equipped with a LC-20AD pump and SPD-M20A detector with a D2&W lamp. To construct the analytical curve, stock solutions of rotenoids were prepared in the concentration range of 5 to 750 μg/mL. Each concentration was analyzed by HPLC under previously established chromatographic conditions. An NST C18 column (250 mm × 4.6 mm; 5 μm) was used as the stationary phase, while the mobile phase consisted of solution A (50% MeOH) and solution B (100% MeOH) as eluents.

For each concentration, two HPLC runs were performed, and the average peak areas obtained were used to construct the analytical curve by linear regression of the data. The wavelength used for absorbance readings was 290 nm. As a result, a calibration curve was obtained with the equation 30875*x* + 108219 (*r* = 0.99399). To determine the concentration of rotenoids in the nanoparticles, a solution containing 1 mg/mL of nanocomposites in 50% MeOH was prepared. The solution was subjected to ultrasonic bath for 10 min at room temperature. Then, the samples were filtered with a 0.22 μm filter and injected into the HPLC system. For each sample, two runs were performed, and the average peak areas obtained were used for concentration calculations.

### Larvicidal activity assay

The larvicidal assay was conducted according to the standards established by the World Health Organization [39] with adaptations [14]. Starting from an initial stock solution (10 mg nanoparticles/mL in water-diluted vinegar [to reach a 0.08% acetic acid concentration]), the nanoparticles were further diluted in ultrapure water to prepare concentrations of 50, 100, and 200 ppm of rotenoids, based on calculations obtained by HPLC. Vinegar, an easily accessible source of acetic acid, was employed because chitosan is a water-insoluble polysaccharide that dissolves only in acidified solutions. Five third-instar larvae were used for 5 mL of each solution, and the tests were performed in triplicate. The negative control was prepared using 0.04% acetic acid vinegar. The larvae were incubated for 24 h at 28 °C. After the incubation period, mortality was assessed by the movement of the larvae, which were also observed under a stereomicroscope. This assay was conducted following the same standards for all four nanoparticle samples: CS/TPP control, CS/TPP–β-CD control, CS/TPP rot, and CS/TPP–β-CD rot.

The mortality data were analyzed using Probit regression performed in RStudio with the glm() function (binomial family, probit link). The lethal concentrations (LC_50_ and LC_90_) were calculated based on the estimated model parameters [40].

### Cellular viability assessment by MTT assay

The cellular viability of human skin fibroblasts (HSF) exposed to rotenoids and to the CS/TPP–β-CD–rot nanocomposite was evaluated using the 3-(4,5-dimethylthiazol-2-yl)-2,5-diphenyltetrazolium bromide (MTT) assay. The cells were cultured in 96-well plates at a density of 3 × 10^5^ cells/mL. After adherence, the cells were treated with either in natura rotenoids, at 250 ppm, or the CS/TPP–β-CD–rot nanocomposite (equivalent to 250 ppm of rotenoids), and incubated for 24 h at 37 °C in a humidified atmosphere with 5% CO_2_. Subsequently, 10 µL of MTT solution (5 mg/mL) were added to each well, and after 2 h of incubation, the supernatant was removed. The resulting formazan crystals were solubilized with acidified isopropanol, and the absorbance was measured at 570 nm using a microplate reader (Thermo Labsystems Multiskan, model 352). A positive control (2% Triton X-100) and a negative control (culture medium without treatment) were used. Cellular viability was expressed as a percentage relative to the negative control. Statistical analysis was performed using one-way ANOVA followed by Tukey’s multiple comparison test (*p* < 0.05) with the GraphPad Prism 10 software.

## Data Availability

All data that supports the findings of this study is available in the published article and/or the supporting information of this article.
